# Comparative Analysis of Cx31 and Cx43 in Differentiation-Competent Rodent Keratinocytes

**DOI:** 10.3390/biom10101443

**Published:** 2020-10-14

**Authors:** Akina Au, Qing Shao, Kyra K. White, Sergiu A. Lucaciu, Jessica L. Esseltine, Kevin Barr, Dale W. Laird

**Affiliations:** 1 Department of Physiology and Pharmacology, The University of Western Ontario, London, ON N6A 5C1, Canada; akina.au@hotmail.com (A.A.); slucaciu@uwo.ca (S.A.L.); 2 Department of Anatomy and Cell Biology, The University of Western Ontario, London, ON N6A 5C1, Canada; cindy.shao@schulich.uwo.ca (Q.S.); kyrawhite@me.com (K.K.W.); Kevin.barr@schulich.uwo.ca (K.B.); 3 Division of BioMedical Sciences, Faculty of Medicine, Memorial University of Newfoundland, St. John’s, NL A1B 3V6, Canada; jesseltine@med.mun.ca

**Keywords:** gap junctions, connexin, Cx31, Cx30, Cx26, Cx43, rat epidermal keratinocytes, differentiation

## Abstract

When considering connexin expression and regulation, the epidermis of the skin is one of the most complex tissues found in mammals even though it largely contains a single cell type, the keratinocyte. In the rodent epidermis, up to 9 connexin family members have been detected at the mRNA level. Many of these connexins are temporally and spatially regulated in coordination with keratinocyte progenitor cell differentiation and migration from the stratum basale to form the stratum spinosum and stratum granulosum layers before finally forming the stratum corneum. Cx43 is the principal connexin found in basal keratinocytes and to a lesser degree found in keratinocytes that have begun to differentiate where Cx26, Cx30 and Cx31 become prevalent. Here we show that the CRISPR-Cas9 ablation of Cx43 reduces overall gap junction coupling in monolayer cultures of rat epidermal keratinocytes (REKs) and dysregulates the differentiation of REKs when grown in organotypic cultures. Natively found in differentiated keratinocytes, Cx31 readily assembles into gap junctions when expressed in REKs where it can extensively co-assemble into the same gap junctions with co-expressed Cx30. Time-lapse imaging indicated that many Cx31 gap junctions are mobile within the plasma membrane undergoing both fusion and fission events. Finally, the persistence of pre-existing Cx31 gap junctions in the presence of the protein trafficking blocker, brefeldin A, is longer than that found for Cx43 gap junctions indicating that it has a distinctly different life expectancy in REKs. Collectively, this study highlights the importance of Cx43 in rodent keratinocyte differentiation and suggests that Cx31 acquires life-cycle properties that are distinct from Cx43.

## 1. Introduction

Gap junctions are specialized membrane channels composed of connexins that allow adjacent cells to directly exchange potentially 3000 members of the metabolome in a process called gap junctional intercellular communication (GJIC) [1,2]. A total of 20 connexin family members are expressed in rodents which proceed to oligomerize into connexons (frequently called hemichannels) *en route* to the plasma membrane [3,4]. At cellular interfaces, hemichannels assemble into intercellular channels that become densely clustered to form gap junctions [5]. The complexity of gap junction channels is immense as most cells express 2 or more connexin subtypes which proceed to selectively assemble into mixed hemichannels composed of different connexins that serve to build sophisticated gap junction communication networks within cells and tissues [6]. Nowhere is this more evident than in the rodent epidermis where keratinocytes express mRNA for up to 9 connexins with Cx26, Cx30, Cx30.3, Cx31, Cx31.1, Cx37 and Cx43 readily expressed as detectable proteins [7,8]. These “keratinocyte connexins” are expressed at variable levels within the different strata of the epidermis to fulfil specific needs as these differentiating keratinocytes act to maintain epidermal homeostasis [8,9,10,11].

Undifferentiated keratinocytes of the stratum basale are enriched in Cx43 which diminishes as keratinocytes proliferate, differentiate and migrate to populate the stratum spinosum and stratum granulosum [8,9,10,11]. Differentiated keratinocytes tend to replace the expression of Cx43 and initiate the expression of any one or more of the other 6 keratinocyte connexins [9]. This reprogramming of connexin expression leads to multiple keratinocytes at various stages of differentiation that simultaneously express 2, 3 or more connexin subtypes. Multiple connexin co-expression in single cells lead to a vast diversity in how channels can be stoichiometrically arranged as cells that express even 2 connexins at the same time can assemble into 196 channel arrangements [4,12,13]. However, not all connexin subtypes interact and pair with each other, raising the question as to what connexins are compatible. Evidence now exists that Cx43 does not typically interact with Cx26 or Cx30 in cells of the epidermis and other tissues. Meanwhile, Cx26 and Cx30 readily co-oligomerize to form gap junctions in several cell types [14,15,16]. Adding to these complexities is the growing evidence that not all connexins have identical half-lives. For example, Cx43 has a rapid half-life of only 1–2.5 h [17,18] but we found that Cx30 has a half-life of >12 h [19], strongly suggesting that the fundamental properties of less studied keratinocyte connexins, such as Cx31, need to be closely considered [7,20].

In Cx31-null mice that survive a placental defect, the epidermis has surprisingly no observable alterations or deficits [21], possibly due to compensatory mechanisms offered by other keratinocyte connexins. Conversely, neonatal mice harboring a truncated C-terminal mutant of Cx43, lacking the domain responsible for much of the Cx43 posttranslational regulation and most of the interactome binding sites, exhibit a severe epidermal barrier defect [22]. Earlier we demonstrated that *sh*RNA-driven partial knockdown of keratinocyte Cx43 led to impaired organotypic epidermis formation suggesting that the ablation of Cx43 may have even more profound effects on the epidermis [23]. Collectively, these in vivo and in vitro findings point to the likelihood that Cx43 and Cx31 serve very different roles in the epidermis and point to a potential essential role for Cx43. The importance of these connexins in the skin is further emphasized by the fact that mutations in the genes encoding both Cx31 and Cx43 can lead to erythrokeratodermia variabilis et progressiva [1,24,25].

In order to study the role, localization, assembly and fate of multiple connexins in rodent keratinocytes, a differentiation-competent rodent cell line is essential. Rat epidermal keratinocytes (REKs) are a valuable model to examine epidermal connexins because, unlike many other epidermal derived cell lines, REKs retain the capacity to differentiate when grown at a liquid/air interface [26]. Mimicking full thickness rat epidermis, the organotypic epidermis is comprised of a basal cell layer, several suprabasal cell layers and a cornified layer. Additionally, REKs express transcripts for a vast complement of epidermal connexins that includes Cx26, Cx30, Cx30.3, Cx31, Cx37, Cx40, Cx43 and Cx45 but only express meaningful protein levels of Cx43 and trace amounts of Cx26 [23,27]. Furthermore, these cells are receptive for the ectopic expression of fluorescent protein-tagged Cx43, Cx30 and Cx26, all of which have been well characterized in various cell types [28,29,30]. In the current study, we employ REKs to interrogate the functional role of Cx43 in keratinocyte differentiation and its potential interplay with Cx31.

## 2. Materials and Methods

### 2.1. Cell Cultures

Rat epidermal keratinocytes (REKs) were characterized by Baden and Kubilus [26] and kindly provided by Dr. Vincent Hascall (Cleveland Clinic, Cleveland, OH, USA). REKs and normal rat kidney (NRK) cells were cultured in Dulbecco’s Modified Eagle Medium (DMEM) (Cat# 11960-044, Life Technologies, Carlsbad, CA, USA) supplemented with 10% FBS, 100 units/mL penicillin-streptomycin and 2 mM L-glutamine (Cat# 25030-081, Life Technologies) and incubated at 37 °C and 5% CO_2_. When 70–90% confluency was reached REKs were passaged using 0.25% trypsin/EDTA (Cat# 325-040-EL, Wisent, Saint-Jean Baptiste, QC, Canada) for 5–10 min at 37 °C and 5% CO_2_. Cells were seeded into 35-mm optical-grade plastic-bottomed dishes (Cat# 81156, Ibidi, Grafelfing, Germany) for live imaging studies, or 60-mm plastic tissue culture dishes containing glass coverslips for immunocytochemistry studies.

Primary mouse keratinocyte cultures were generated from the dorsal skin of newborn C57BL/6 mice, as previously described [31,32]. Frozen sections of skin were prepared from adult mice as described [33] and used as a positive control for anti-connexin labeling. All mouse studies were approved by the Animal Care Committee at the University of Western Ontario (Protocol # 2015-030; 2019-009) in keeping with the Canadian Council of Animal Care.

### 2.2. cDNA Constructs, Transfections and CRISPR-Cas

The construct encoding Cx31-GFP was a kind gift from Dr. Gabriela Richard (Thomas Jefferson University, Philadelphia, PA, USA). cDNA encoding Cx26 and Cx30 were cloned into pEGFP-N1 vectors (BD Biosciences Clontech, Palo Alto, CA, USA) or pTagRFP-N vectors (Evrogen; Cedarlane, Burlington, ON, Canada) as described previously [28]. Rat Cx43-GFP was previously described [29,34]. The GFP or RFP tags do not noticeably affect connexin trafficking or gap junction formation as previously demonstrated [28,29,30,35].

For cell transfections, approximately 400,000 REKs were seeded into 60-mm dishes with coverslips and transiently transfected the following day with 1 μg of DNA using lipofectamine 3000 (Cat# L3000001, Invitrogen, Carlsbad, CA, USA). For each dish, a two-reaction mixture was used during the transfection: reaction 1 mixture consisted of 125 μL Opti-MEM Reduced Serum Medium (Life Technologies Cat# 31985-070) and 3.7 μL Lipofectamine 3000 reagent; reaction 2 mixture consisted of 250 μL Opti-MEM Reduced Serum Medium, 0.5–1 μg of DNA and 4 μL lipofectamine 3000 reagent. Reaction 1 and 2 mixtures were combined, gently mixed, incubated for 10–15 min at room temperature and added dropwise to cells growing in DMEM. For co-transfections, 0.5 μg of DNA for each construct was used (1 μg total). All subsequent experiments were performed 24–48 h post-transfection.

To examine the role of Cx43 in REKs, the *Gja1* gene encoding rat Cx43 was ablated using CRISPR-Cas9 following strategies we previously described [36,37]. Briefly, cells successfully expressing the gRNA were single-cell fluorescence activated cell sorted and plated for single colony cell growth. Approximately 1 week later, surviving cells were sub-cultured into a 25 cm^2^ flasks for further expansion. Three Cx43-ablated clones (designed C1, C2 and C3) were generated and Cx43 ablation was confirmed through Western blotting and immunolabeling for Cx43. Since no differences in cellular morphology or keratin 14 distribution was observed between all three Cx43-ablated clones, in some cases, we combined these clones to form a Cx43-ablated multi-clone cell line.

### 2.3. Organotypic Cultures

Organotypic epidermis was grown on 24 mm transwell filter inserts (BD Biosciences, San Jose, CA, USA) with a 3.0 µm pore size as we have described [23,27,38]. Briefly, inserts were coated with 1.5 mL of collagen I substrate (8.5 mL of type I collagen, 1 mL 10× HBSS (Hanks balanced salt solution) with Phenol Red (MilliporeSigma, Burlington, MA, USA), 100 µL 1.0 M HEPES and 200 µL 1 M NaOH). Collagen substrate was polymerized by incubating at room temperature for 2 h. Following polymerization, the collagen layer was washed 3× with PBS and 2 mL of REK growth media was added to the top chamber and left overnight at 37 °C/5% CO_2_. The following day the top media was changed and 2 mL of media was added to the bottom chamber. REKs and Cx43- knockout (Cx43 KO) REKs were plated dropwise in the upper chamber at a density of 500,000 cells/2 mL media/well insert. Cells were grown to confluency over a 3-day period, while media was changed daily in the top and bottom chambers. Once confluent, media was removed from the upper chamber to create the air-liquid interface to promote cell differentiation. Cells were cultured for an additional 21 days and lower media was changed daily.

For morphometric analysis, organotypic epidermis was harvested by peeling the epidermal culture off the collagen matrix and fixed in 10% formalin for 1 h at room temperature. One quarter of harvested epidermal cultures were processed for paraffin embedding. Samples were sectioned at 5 µm thickness. Sections of organotypic epidermis were deparaffinized and stained with hematoxylin and eosin. Images were collected using a 40× objective on an EVOS™ XL Core configured microscope (Cat#. AMEX1100, ThermoFisher, Waltham, MA, USA). The cornified thickness and vital cell layer thickness were measured at 6 random regions across 5 images using ImageJ.

For immunolabeling studies, one quarter of harvested organotypic epidermis was suspended in Optimal Cutting Temperature compound (Cat# 4583, Tissue-Tek, Maumee, OH, USA) and frozen at −80 °C until sectioned. Cryosections were fixed with 10% formalin for 1 h at room temperature and immunolabeled as described below. A Zeiss LSM 800 confocal microscope and 63× oil immersion objective were used for imaging. Using ZEN Blue software, Z-stack orthogonal projections flattened to 2D images were used to capture the full 20 µm immunolabeled sections, imaged at 1.0–1.3 µm increments.

### 2.4. Immunocytochemistry and Immunofluorescent Imaging

Cells and cryosections were fixed, permeabilized and blocked with 0.1% Triton X-100 in 2% bovine serum albumin (Cat#A2153, Sigma) in phosphate-buffered saline (PBS) for 1 h at room temperature. The primary antibodies used included: 1:100 rabbit polyclonal Cx31 antibody (Thermo Fisher, Waltham, MA, USA 36-5100), 1:400 rabbit polyclonal anti-Cx26 (Cat# 51-2800, Life Technologies), 1:400 rabbit polyclonal anti-Cx30 (Cat# 71-2200, Life Technologies), 1:500 rabbit polyclonal anti-Cx43 (Cat# C6219, Sigma-Aldrich, St. Louis, MO, USA), mouse anti-Cx43 (1:50, P4G9), 1:100 mouse anti-keratin 14 (NeoMarkers Freemont, CA, USA, LL002), 1:200 mouse monoclonal anti-cytokeratin 14 (Cat# MS-115-P, Thermo Fisher Scientific, Waltham, MA, USA), 1:200 rabbit polyclonal anti-keratin 10 (Cat# PRB-159P, Covance, Princeton, NJ, USA) and 1:500 mouse monoclonal anti-GM130 (Cat# 610822, BD Transduction Laboratories, San Jose, CA, USA). The secondary antibodies used were a 1:500 dilution of Alexa-488-conjugated anti-mouse (Cat# A11017, Life Technologies) and a 1:500 dilution of Alexa-555-conjugated anti-rabbit (Cat# A-21428, Thermo Fisher Scientific, Waltham, MA, USA). Nuclei were stained with Hoechst 33342 (Cat# H3570, Molecular Probes, Eugene, OR, USA), mounted with Airvol and imaged using a Zeiss LSM 800 confocal Airyscan microscope equipped with ZenWorks software (Newbury, Berkshire, UK). Images were acquired using a 40× oil or 63× oil immersion objective. As positive controls for anti- Cx30, Cx26 and K10 antibody labeling, REKs expressing Cx30-RFP, NRK cells expressing untagged Cx26 and wild type mouse epidermis were used, respectively. All positive control samples were subsequently detected using Alexa-488-conjugated secondary antibody.

### 2.5. Connexin Co-Localization Analysis

REKs co-expressing Cx31-GFP + Cx26-RFP, Cx31-GFP + Cx30-RFP and Cx31-GFP + Cx43-RFP were imaged using a 63× oil immersion objective on a Zeiss LSM 800 confocal microscope. Images were acquired through 15 Z-stack optical slices with each slice being acquired at approximately 0.2 μm intervals (approximate intervals were determined through the ‘optimal section’ function in Zeiss software, (Oberkochen, Germany). Image stacks were superimposed for co-localization analysis. Use of the ‘range indicator’ function ensured that all gap junctions were captured with negligible pixel saturation. Using the ZenWorks co-localization software, regions of interest (ROIs) were drawn around each gap junction and a scatterplot of red and green pixels (representing the 2 co-expressed connexins) were generated. The Coste’s algorithm generated an automatic threshold and the Pearson’s correlation coefficient (PCC), a statistic for quantifying colocalization, was computed. A minimum of 60 gap junctions were analyzed for each connexin pair and a one-way ANOVA was performed on the means of 3 biological replicates.

### 2.6. Live Cell Time-Lapse Imaging

Approximately 200,000 REKs were seeded into 35-mm optical grade plastic-bottomed dishes (Cat# 81156, Ibidi) and transfected the following day with 0.5 μg of Cx31-GFP encoding plasmid. Approximately 24 h post-transfection, dishes were mounted onto a Zeiss LSM 800 confocal microscope within a 37 °C heated chamber containing 5% CO_2_. Using a 63× oil immersion lens, images were acquired every ~20–30 s for up to a period of 30 min following general procedures as we previously described [29]. Cx31-GFP was excited with a 488 nm argon laser and emitted fluorescence was collected through a 500–550 nm band pass filter. Fluorescent images and differential interference contrast (DIC) images were acquired simultaneously and superimposed. A minimum of 40 Cx31-GFP gap junctions were examined in live cells. 

### 2.7. Cell Lysates and Immunoblotting

Cell lysates were collected with 2x IP lysis buffer (2% Triton X-100, 300mM NaCl, 20mM Tris, 2mMethylenediaminetetraacetic acid, 2mMethylene glycol-bis(β-aminoethyl ether)-N,N,N,N-tetraacetic acid, 1% NP-40, pH 7.4) containing 100 mM NaF, 100 mM sodium orthovanadate and a proteinase inhibitor mini-EDTA tablet. Protein content was quantified using a bicinchoninic acid assay kit (Cat# 23225, Thermo Fisher Scientific, Waltham, MA, USA). Protein (25–30 μg) was loaded in each lane, separated by 10% SDS-PAGE and transferred onto nitrocellulose membranes using the iBlot^®^ dry-transfer system (Invitrogen, Carlsbad, CA, USA). Membranes were labeled with 1:5000 dilution of rabbit polyclonal anti-Cx43 (Cat# C6219, Sigma-Aldrich) and 1:5000 dilution of mouse anti-glyceraldehyde-3-phosphate dehydrogenase (GAPDH) (Cat# MA374, MilliporeSigma, Burlington, MA, USA). The following secondary antibodies used were as follows: a 1:10,000 dilution of goat anti-mouse Alexa Fluor 680 (Cat#A21057, Life Technologies) and a 1:10,000 dilution of goat anti-rabbit IRDye 800 (Cat# 611-132-002, Rockland Immunochemicals, Pottstown, PA, USA). Protein bands were visualized using Odyssey LiCor infrared imaging system.

### 2.8. Scrape Load Assay

REKs with or without endogenous Cx43 were loaded with 1.5 mg/mL Lucifer yellow (457 Daltons) (Sigma, L0259-25MG) and 0.5 mg/mL dextran rhodamine B (10,000 Daltons, Thermo Fisher D1824) in PBS. Using a blade, plates were scraped in 3 distinct locations, then incubated at 37 °C/5% CO_2_ for 3 min. The dye was removed, cells were washed 3× with PBS and fixed with 10% formalin for 15 min at room temperature. Cells were then imaged (3 images per scrape) using a Zeiss LSM 800 confocal Airyscan microscope equipped with a 10× objective. Lucifer yellow dye transfer was quantified using ImageJ via measuring the distance from the first row of ruptured cells. A total of 6 measurements were taken at 3 distinct points along each scrape with a total of 4 biological replicates per cell line.

### 2.9. Fluorescence Recovery after Photobleaching

Wildtype and Cx43 KO REKs were seeded into 35-mm glass bottom dishes (Cat# 10810-056, VWR, Mississauga, ON, Canada) and incubated for 24 h at 37 °C and 5% CO_2_. A stock dye solution was prepared by dissolving 50 µg of CellTrace™ Calcein Red-Orange, AM (Cat# C34851, ThermoFisher) in 10 µl DMSO and stored at −20 °C. For each dish, 1 µL of the stock calcein solution was added to 1 mL Opti-MEM Reduced Serum Medium. Growth media was aspirated, cells were loaded with calcein Red-Orange AM dye for 10 min at 37°C and 5% CO_2_. Cells were subsequently washed three times with PBS and replenished with room temperature Opti-MEM. Dishes were transferred to a live cell imaging apparatus maintained at 37 °C for subsequent imaging. Single cells within small cell clusters were randomly selected and imaged prior to bleaching using a Zeiss LSM 800 confocal Airyscan microscope equipped with a 63× oil immersion objective lens. Cells were photobleached to ~20% of the initial fluorescence intensity using a 561-nm argon laser. Fluorescence recovery was imaged at 1 s intervals for 2 min and quantified using the Time Series Analyzer v3 plugin for ImageJ (Los Angeles, CA, USA). The proportion of fluorescence recovery was calculated by dividing the difference between the fluorescence intensity at each time point and the fluorescence intensity immediately after bleaching by the initial fluorescence intensity prior to bleaching. Fluorescence recovery was plotted as a function of time and the area under the curve (AUC) was calculated for each bleached cell. A total of 37 Cx43-ablated REKs and 38 wildtype REKs were analyzed. AUC data were analyzed for outliers using the robust regression and outlier removal method on GraphPad Prism 8.3.4. A total of five outliers were identified and removed in each group. AUC data were then analyzed using Mann-Whitney test on GraphPad Prism. (San Diego, CA, USA).

### 2.10. Drug Treatment

To block ER-Golgi protein transport, Cx31-GFP expressing wild type REKs growing on glass coverslips were treated with 4 μg/mL of brefeldin A (BFA) (Cat# B5936, Sigma) in DMEM for up to 6 h at 37 °C and 5% CO_2_ prior to BFA washout and a further 2 h incubation period in DMEM. All cells were fixed and immunolabeled for Cx43 and GM130 as well as nuclei were stained with Hoechst 33342 dye. A minimum of 5 images were acquired on a Zeiss LSM 800 microscope at each treatment time point and the number of cell-cell interfaces that expressed Cx43 and Cx31 gap junctions were quantified for each treatment where the researcher was blinded to the treatment. Only Cx31-GFP positive cell pairs or clusters were considered; a minimum of 75 cell-cell interfaces were quantified and a two-way ANOVA was performed on the means of 3 biological replicates.

## 3. Results

### 3.1. Monolayer REKs Are Enriched in Cx43 and Make Prototypical Gap Junctions

**Figure 1:**
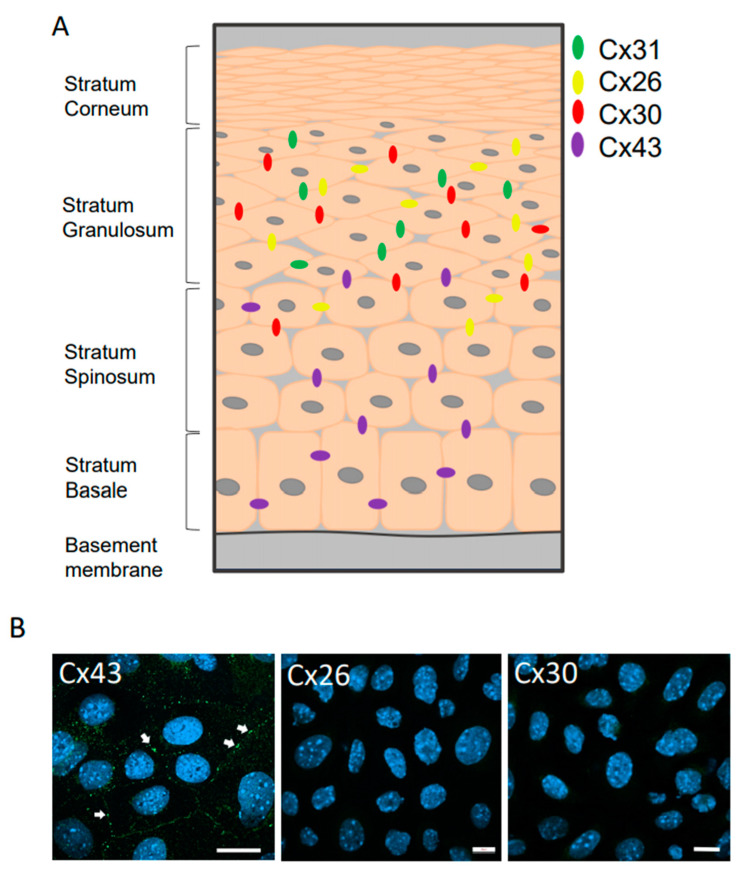
(**A**) Schematic model denoting the approximate location of Cx26, Cx30, Cx31 and Cx43 expression in rodent epidermis. Note that Cx43 is found primarily in the basale and spinosum strata while Cx26 and Cx30 are more commonly found in the upper spinosum strata and throughout the stratum granulosum. Cx31 is typically restricted to the stratum granulosum. At least three other connexins are expressed in the epidermis that are not shown here. (**B**) Primary keratinocytes from newborn mice were immunolabeled to detect the presence of Cx43, Cx26 and Cx30. Note that while clearly identifiable Cx43 gap junctions were observed (arrows) no Cx26 or Cx30 could be detected. Bar = 10 μm.

While rodent epidermis contains 7 detectable connexin protein subtypes, Figure 1A depicts the approximate localization profile of the 4 that are most often identified in keratinocytes of the epidermis. It is noteworthy that Cx43 is widely found in the stratum basale with decreasing prominence in keratinocytes that have differentiated and migrated into other strata. Conversely, Cx26, Cx30 and Cx31 are more conspicuously found within differentiated keratinocytes with Cx31 being more restricted to the stratum granulosum [39]. When primary keratinocytes rich in basal cells were cultured from newborn mouse epidermis, Cx43 gap junctions were readily detectable but no Cx26 or Cx30 was observed (Figure 1B).

**Figure 2:**
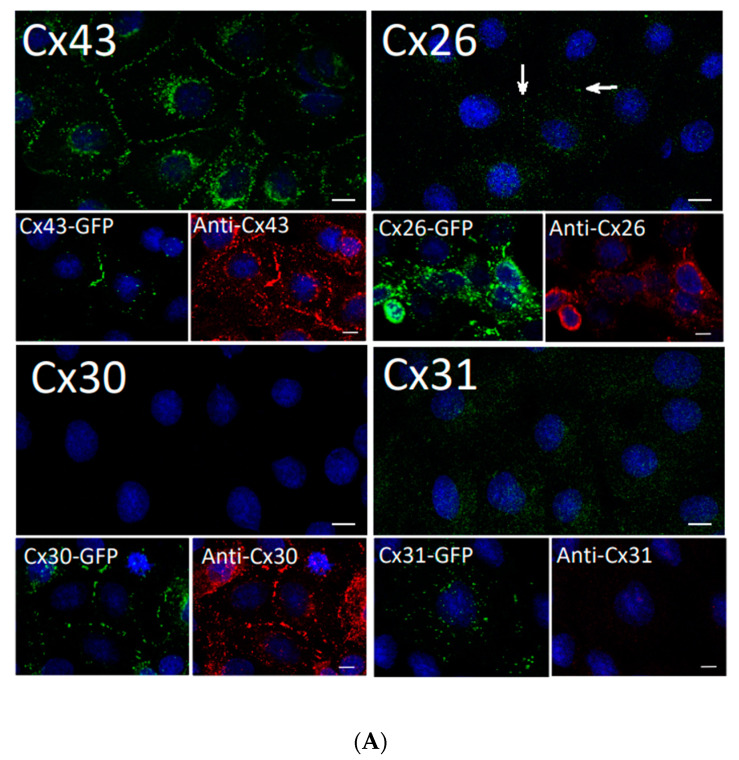

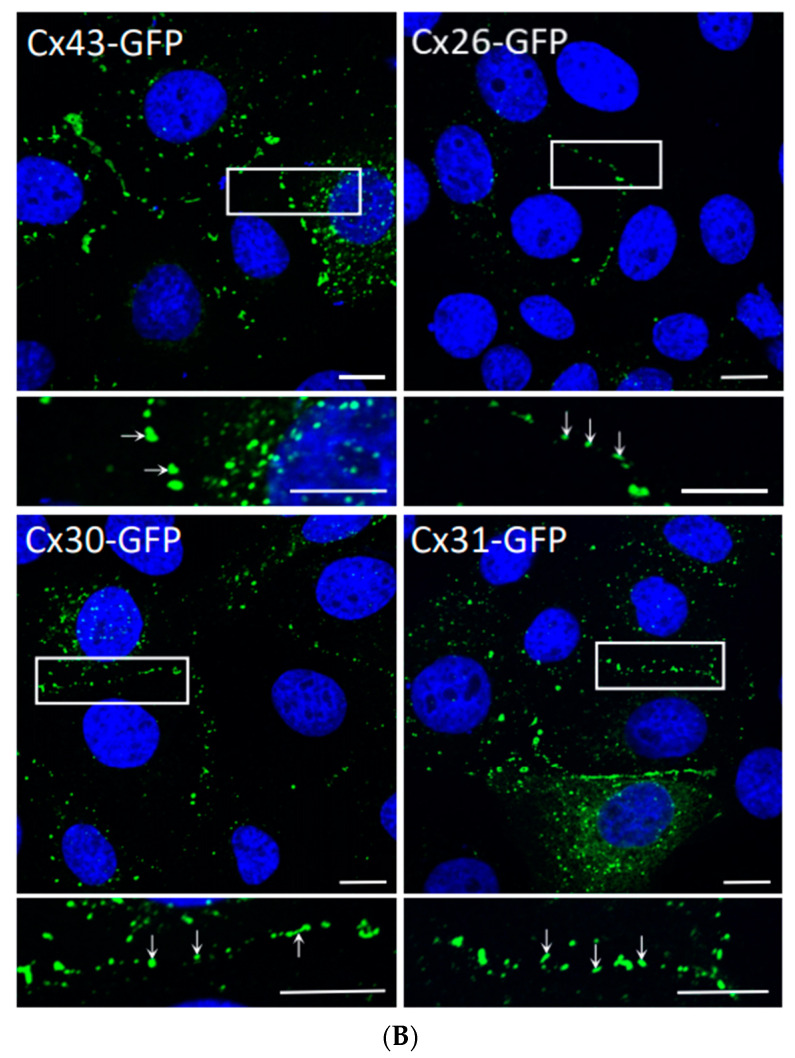
Rat epidermal keratinocytes (REKs) express endogenous Cx43 and retain the capacity to assemble GFP-tagged Cx26, Cx30 and Cx31 into gap junctions. (**A**) Basal keratinocyte-like REKs express endogenous Cx43, trace amounts of Cx26 (arrows) but no detectable levels of Cx30 or Cx31 protein. REKs engineered to express GFP-tagged connexins were used to demonstrate that the anti-connexin antibodies were efficient at detecting connexins with the exception of the anti-Cx31 antibody. (**B**) REKs engineered to express GFP-tagged Cx43, Cx26, Cx30 and Cx31 all assembled prototypical gap junctions characterized by small punctate structures at sites of cell-cell interface (arrows). In addition, an array of intracellular fluorescent structures could be identified that reflect organelles and vesicles involved in connexin transport. Nuclei were stained with Hoechst dye. Bars = 10 μm.

Since rat epidermal keratinocytes (REKs) were spontaneously immortalized from cell cultures enriched in basal keratinocytes and were differentiation-competent [26], we characterized the endogenous connexins expressed in monolayers of REKs (Figure 2A). Numerous Cx43 gap junctions were detected at cell-cell appositions, mimicking the Cx43 status found in primary mouse keratinocytes (Figure 1B). Only rare and small Cx26 gap junctions were detected in REKs (Figure 2A, arrows) consistent with our previous findings [27]. No Cx30 gap junctions were detected at cell-cell appositions, unlike positive control cells that were engineered to express GFP-tagged Cx30. No antibody labeled Cx31 gap junctions were detected in REKs or the positive control (Figure 2A, inserts), indicating the anti-Cx31 antibody used was ineffective. To circumvent the inability to examine endogenous Cx31 in REKs, we chose to examine the life cycle of Cx31 by tagging it with GFP (Cx31-GFP) and ectopically expressing it in REKs. Cx31-GFP localized to punctate structures, reminiscent of gap junctions, at cell-cell appositions (Figure 2B). To compare and confirm that Cx31-GFP formed punctate structures similar to other well characterized GFP-tagged connexins [28,29,30,35], GFP-tagged Cx30, Cx26 and Cx43 were expressed in REKs. As expected, Cx30-GFP, Cx26-GFP and Cx43-GFP all formed gap junction plaques (Figure 2B). Together, we show the ectopically expressed Cx26-GFP, Cx30-GFP, Cx31-GFP and Cx43-GFP all form prototypical gap junctions in REKs.

### 3.2. Cx43 Ablation had NO Effect on REKs in 2D Cultures but Grossly Dysregulated Differentiation in Organotypic Cultures

**Figure 3:**
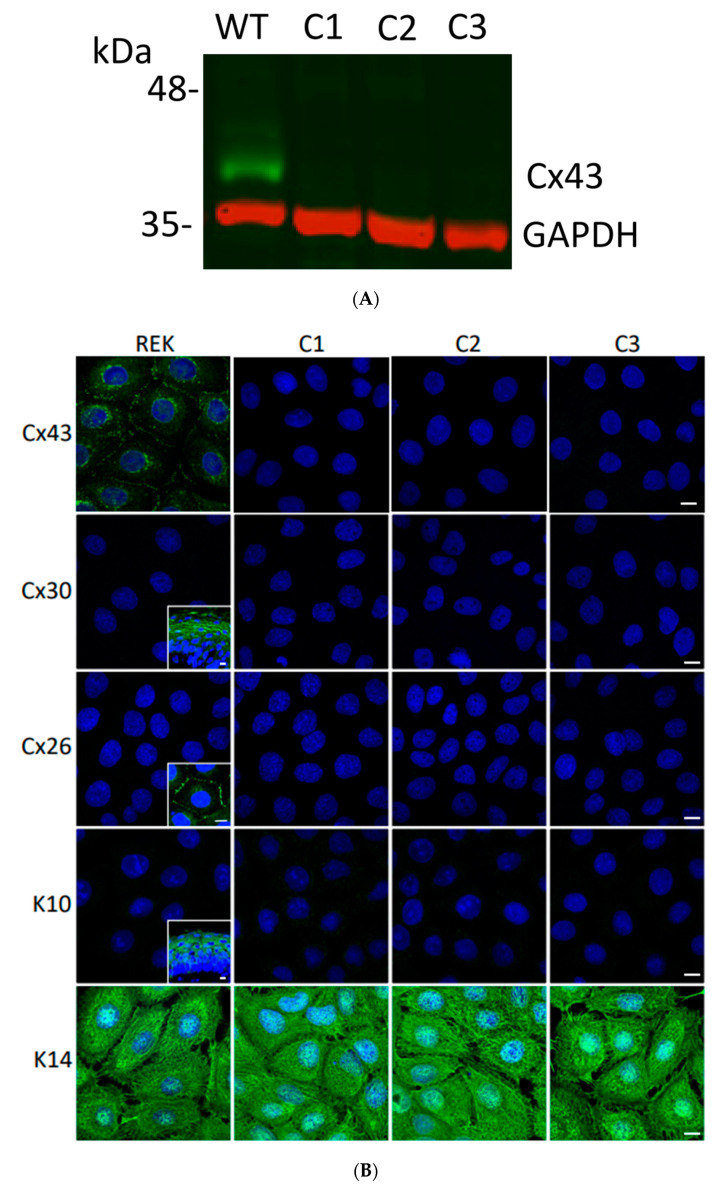
Ablation of Cx43 did not act to stimulate connexin reprogramming or differentiation of REKs. (**A**) CRISPR-Cas9 was effectively used to ablate Cx43 from REKs as revealed by immunoblotting wild-type (WT) and three separate clones (C1, C2, C3) for Cx43. Immunoblotting for GAPDH was used as a loading control. (**B**) Immunofluorescent labeling for Cx43, Cx26 and Cx30 revealed that Cx43 was ablated from all three CRISPR-Cas9 treated clones and neither Cx26 nor Cx30 exhibited increased expression to compensate for the loss of Cx43. Immunolabeling for keratin 10 (K10) and keratin 14 (K14) revealed that REKs retained their basal cell characteristics upon Cx43 ablation. Inserts represent transverse or *en face* sections of mouse skin as positive controls for anti-Cx30 and anti-K10 antibodies while normal rat kidney cells engineered to express Cx26-GFP were used as a positive control for the anti-Cx26 antibody. Nuclei were stained with Hoechst dye. Bars = 10 μm.

Since Cx43 is highly expressed within REKs, we assessed whether Cx43 ablation would drive the compensatory expression of other keratinocyte connexins, specifically, Cx26 or Cx30. Using CRISPR/Cas9 strategies we ablated Cx43 in three separate REK clones as validated by immunoblotting for Cx43 (Figure 3A). In Cx43-ablated REKs, immunofluorescent labeling for Cx26 and Cx30 revealed that their expression was not induced (Figure 3B). To assess whether the complete ablation of Cx43 would trigger keratinocyte differentiation in monolayer cultures, we immunolabeled Cx43-ablated REKs for keratin 10 (K10) (indicative of more differentiated cells) and K14 (indicative of undifferentiated cells). K10 was not detected in our Cx43-ablated REKs, similar to wild type REKs (Figure 3B). Furthermore, the immunolabeling pattern of K14 in Cx43-ablated REKs was unaltered with no apparent change in cell size or morphology. Thus, the loss of Cx43 did not trigger either the expression of Cx26/Cx30 or promote the differentiation state of REKs when maintained in monolayer cultures.

**Figure 4:**
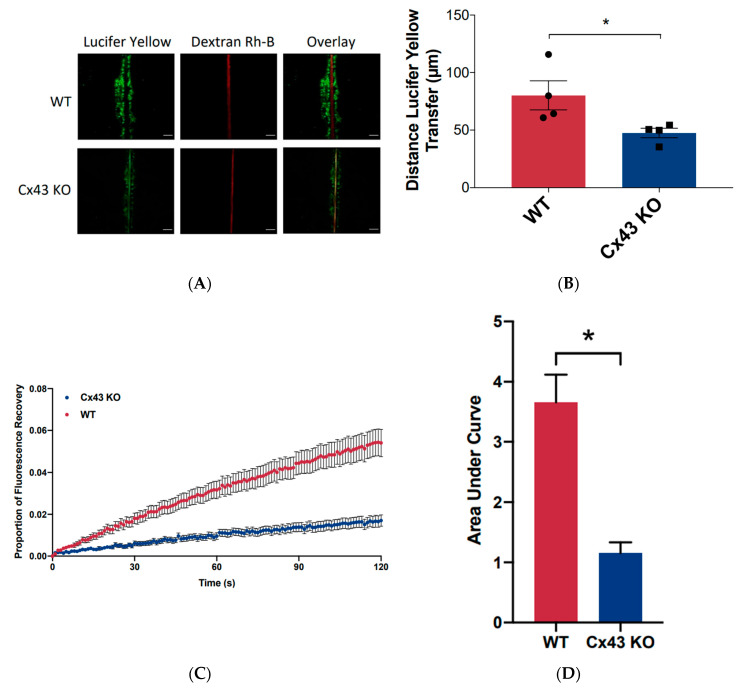
Cx43 ablation from REKs significantly reduced gap junctional intercellular communication. (**A**) Monolayers of wild-type (WT) REKs and REKs where Cx43 was knocked out (Cx43 KO) were scraped and loaded with Lucifer yellow and dextran-rhodamine B (Rh-B) to assess dye spread. Note that Lucifer yellow spread extensively from the wounded edge of WT cells but far less in Cx43 KO cells while dextran Rh-B was only found at the wound edge in both cases. (**B**) Quantification of dye transfer revealed that Cx43 KO cells were far less coupled by gap junctions compared to WT cells. Solid circles represent scattered WT points while solid squares represent scattered Cx43 KO points. (**C**) REKs and Cx43 KO REKs were loaded with calcein AM prior to one cell being laser photobleached. Fluorescence recovery mediated by dye transfer into the photobleached cell was tracked over 120 s. (**D**) Quantification of the area under the curve reflects that Cx43 KO exhibit less GJIC than WT REKs. * *p* < 0.05, data plotted as ± standard error of the mean (SEM), Bar = 10 μm.

To assess if Cx43 ablation notably diminished the overall level of gap junctional intercellular communication (GJIC), wild type (WT) REKs and REKs where Cx43 was knocked out (Cx43 KO) were subjected to a scrape loading dye transfer assay (Figure 4A,B). While dextran rhodamine B demarked the scrape damaged cells, Lucifer yellow spread extensively from the scrape edge to many undamaged WT REKs. However, Cx43 KO cells exhibited significantly reduced dye transfer but dye transfer was not eliminated (Figure 4A,B) suggesting that residual keratinocyte connexins were able to salvage some GJIC. These findings were confirmed by performing a fluorescence recovery after photobleaching assay that assessed the movement of calcein AM into the photobleached cell (Figure 4C,D). Similarly, Cx43 KO cells exhibited significantly reduced dye transfer compared to WT REKs.

**Figure 5:**
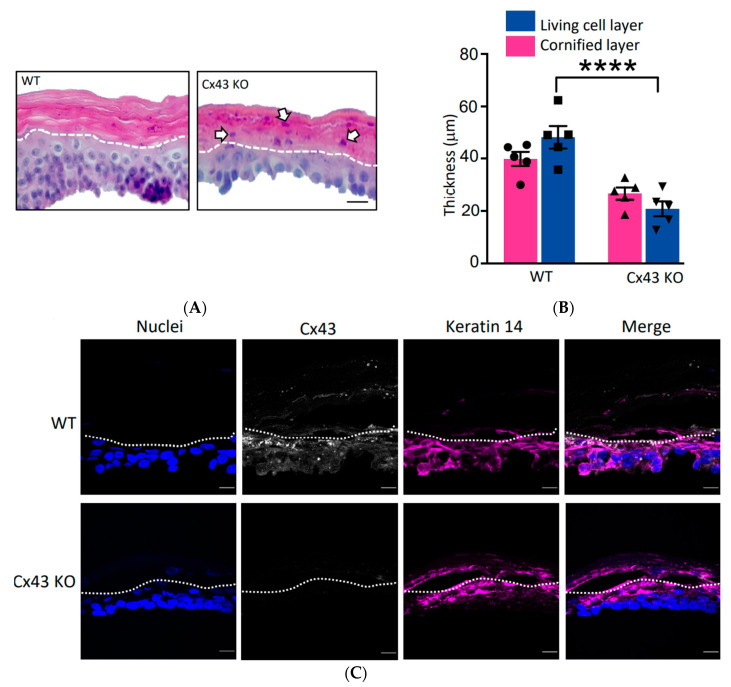

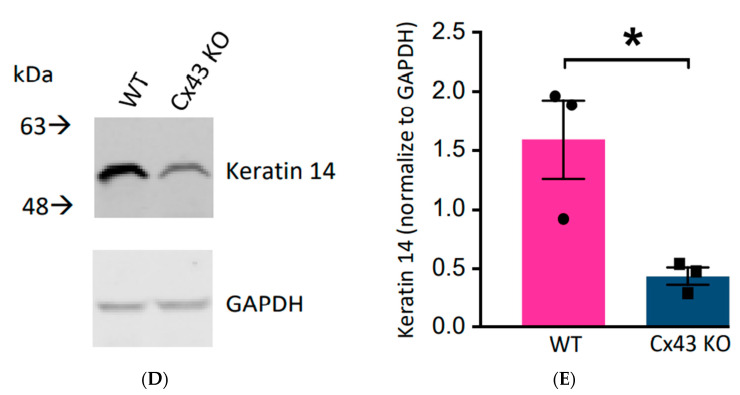
Ablation of Cx43 impairs REKs from fully differentiating in 3-dimensional cultures. (**A**) Wild-type (WT) and Cx43 knock out (Cx43 KO) REKs were grown at a liquid-air interface for three weeks and stained with hematoxylin and eosin. Note that the Cx43 KO epidermis cultures were thinner and many more nuclei were trapped within the cornified layers (arrows) above the dashed line. Bar = 20 μm. (**B**) Quantification of the artificial epidermis thickness revealed that the living cell layer was remarkably thinner when Cx43 KO cells were used. **** *p* < 0.001. Solid circles represent scattered measures of the WT cornified layer while solid squares represent scattered measures of the WT living cell layer. Right-side up triangles represent scattered measures of the Cx43 KO cornified layer while upside-down triangles represent scattered measures of the Cx43 KO living cell layer. (**C**) Immunolabeling for Cx43 and keratin 14 revealed that there was a delay in the Cx43 KO cells in silencing keratin 14 expression and/or its degradation as this cytokeratin persisted in the cornified layer (above the dotted line). Nuclei were stained with Hoechst dye. Bar = 20 μm. (**D**,**E**) Immunoblotting and quantification revealed that total keratin 14 levels were significantly reduced in Cx43 KO epidermis cultures when normalized to GAPDH. * *p* < 0.05, data plotted as ± SEM. Solid circles represent scattered measures of WT samples while solid squares represent scattered measures of Cx43 KO samples.

Previous evidence in vitro [23] and in vivo [22] suggested that the ablation of Cx43 might entirely eliminate the ability of REKs to form organotypic epidermis. In our experiments here, we found that when wild type (WT) REKs and Cx43 KO keratinocytes were grown as organotypic epidermis, both cultures were able to form multiple living cell layers and cornify, but the Cx43 KO cells were severely compromised (Figure 5A). First, the living cell layer of Cx43 KO organotypic cultures was notably and quantifiably thinner (Figure 5A,B). Second, many keratinocyte nuclei were trapped in the cornified layer in a process known as parakeratosis (Figure 5A, arrows). Third, immunofluorescent labeling revealed that keratin 14 persisted deep into the differentiated cell layer, even being present in the cornified layer, suggesting dysregulation of keratin 14 gene expression and/or its degradation (Figure 5C). Finally, since the images of keratin 14 labeling were saturated and not suitable for quantification, immunoblotting revealed that the overall level of keratin 14 was significantly reduced (Figure 5D,E). Collectively, these studies clearly show that the ablation of Cx43 does not eliminate the ability for REKs to differentiate in 3D cultures but this process is grossly dysregulated.

### 3.3. Cx31 Trafficking and Gap Junction Assembly Occurs Independent of the Cx43 Status

**Figure 6:**
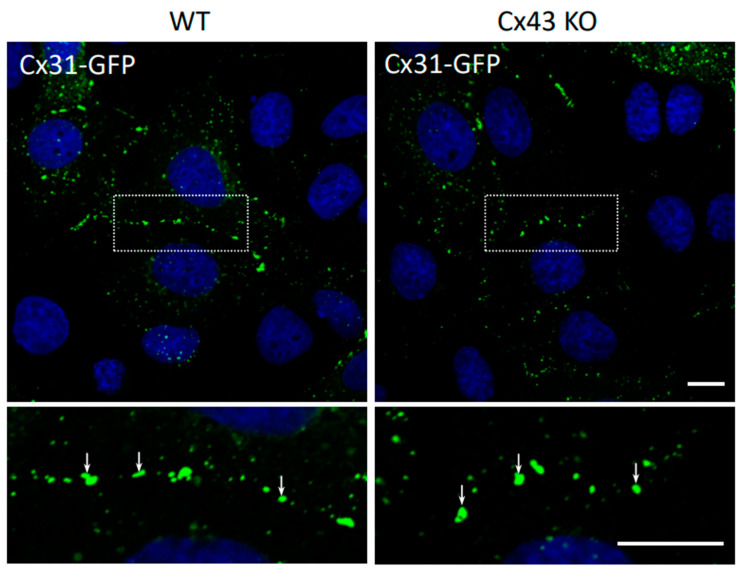
Cx31 assembles into gap junctions independently of Cx43. Wild-type (WT) REKs and REKs lacking Cx43 (Cx43 KO) were engineered to express Cx31-GFP. Note that both cell lines were efficient at assembling Cx31 gap junctions (arrows) as revealed by low and high magnification images. Nuclei were stained with Hoechst dye. Bar = 10 μm.

Since connexin knockout mouse studies suggest that Cx43 elimination is incompatible with survival [40] and Cx31 ablation has no obvious effect on the epidermis in surviving mice [21], we address the possibility that these two connexins may have little interplay or crosstalk during their cellular life cycles. First, we assessed whether Cx31-GFP assembly was notably different in REKs that contained endogenous Cx43 from those where Cx43 was ablated (Figure 6).

**Figure 7:**
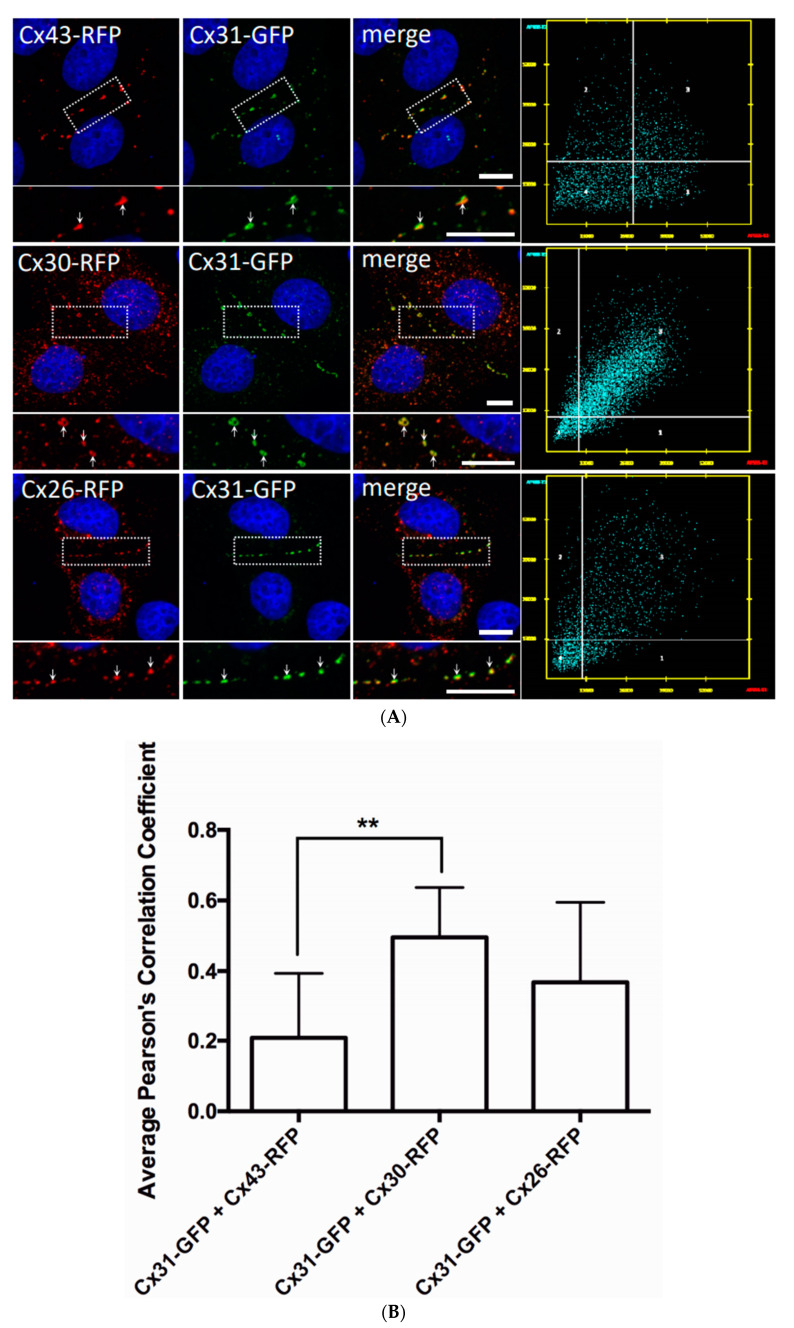
Cx31 colocalized better with Cx30 compared to Cx43. (**A**) REKs were engineered to co-express Cx31-GFP with either Cx43-RFP, Cx30-RFP or Cx26-RFP and assessed for colocalization using the Pearson’s correlation coefficient. Higher magnification inserts images revealed that Cx31 was co-assembling into gap junctions that also contained Cx30 and Cx26 (note yellow, arrows) while Cx43 more often appeared adjacent to Cx31 gap junction plaques (arrows). Nuclei were stained with Hoechst dye. Bars = 10 μm. (**B**) Quantification of colocalization patterns revealed that Cx31 was significantly more colocalized with Cx30 than with Cx43. ** *p* < 0.01, data plotted as ± SEM.

In both cases, many prototypical Cx31-GFP gap junctions were assembled suggesting that the presence or absence of Cx43 did not impact the assembly status of Cx31. To determine if Cx43 could co-assemble into the same gap junctions as Cx31, we co-expressed Cx31-GFP with Cx43-RFP and examined if these connexins co-localized (Figure 7A). Cx31-GFP was infrequently found at identical sites as Cx43-RFP suggesting that these connexins typically assembled into gap junctions that were in proximity to each other, but distinctly separated. That said, we chose to compare Pearson’s correlation coefficients of Cx31-GFP/Cx43-RFP, to other connexin co-expression scenarios and found that Cx31-GFP had statistically better colocalization with Cx30-RFP while Cx31-GFP colocalization with Cx26-RFP did not meet statistical significance (Figure 7B). These data point to the probability that Cx31 and Cx30 can readily co-assemble into the same gap junctions but does not preclude the possibility of Cx31 also sharing the same gap junction plaques with Cx26 as suggested by Di et al. (2005) [8] (Figure 7B).

### 3.4. Cx31 Gap Junctions Are Dynamic and Persist Longer at the Cell Surface Compared to Cx43

**Figure 8:**
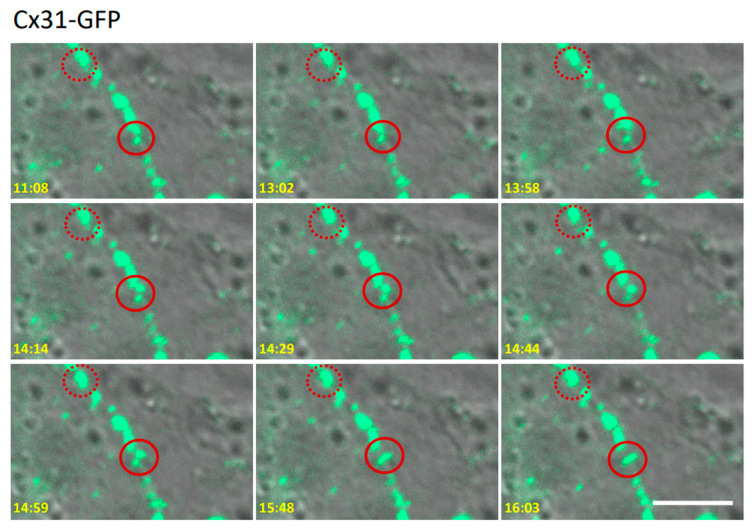
Time-lapse imaging revealed that Cx31-GFP gap junctions were motile at cell-cell interfaces undergoing both plaque fusion and fission. Time-stamped images are presented to denote how some gap junctions remained static over 5 min of time-lapsed imaging (dashed circle) while others (solid circle) underwent rearrangement involving apparent fission and fusion events. Bar = 10 μm.

It has been well established that the long C-terminal domain of Cx43 enables its interaction with an interactome that now exceeds fifty molecules. Some of these molecules serve to interface Cx43 gap junctions with components of the cytoskeletal system, structural junctions and molecular machinery associated with connexin internalization [41,42]. Conversely, to date only a few candidate binding partners have been identified for Cx31, which includes other connexin isoforms [8]. This raises questions as to whether Cx31 displays distinctly different mobility properties within gap junction plaques and how long individual Cx31 gap junctions reside at the cell surface. Time-lapse imaging of Cx31-GFP over a 6-min period revealed that REKs were active in remodeling many gap junctions through a series of fission and fusion events (see Figure 8, solid circle for example).

**Figure 9:**
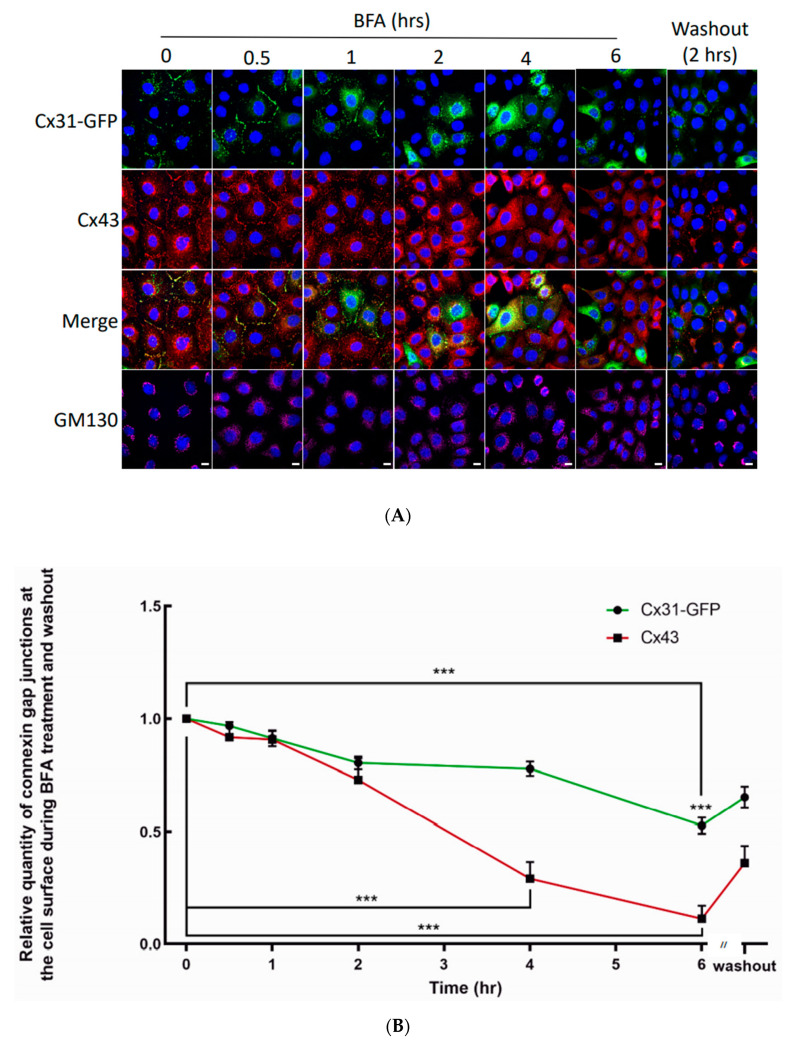
Cx31 gap junctions exhibit a longer lifetime on the cell surface when directly compared to Cx43 gap junctions. (**A**) REKs engineered to express Cx31-GFP were treated with BFA for up to 6 h prior to its removal and a further 2 h incubation. All cells were immunolabeled for Cx43 or GM130 to assess their intracellular distribution. Nuclei were stained with Hoechst dye. Bars = 10 μm. (**B**) Quantification of Cx43 and Cx31-GFP gap junction plaques was assessed during BFA treatment and after BFA washout. Note that Cx31-GFP plaques persisted longer in BFA-treated cells when directly compared to Cx43. Likewise, Cx31-GFP plaque re-assembly appeared to be slower than that of Cx43 upon removal of BFA. *** *p* < 0.001, data plotted as ± SEM.

Other Cx31 gap junctions remained stable and served as an imaging reference point to eliminate cell or image drift during the time course of the analysis (Figure 8, dashed circle). To determine if Cx31-GFP gap junctions might have a longer residence than Cx43 gap junctions at the cell surface, Cx31-GFP expressing cells were imaged for 6 h when protein transport was blocked with brefeldin A (BFA) and for a further 2 h after BFA removal (Figure 9). Parallel immunolabeling for endogenous Cx43 revealed that Cx43 gap junctions largely disappeared within 4 h of BFA treatment while Cx31-GFP plaques remained abundant (Figure 9A,B).

The rapid loss of Cx43-GFP gap junction in BFA-treated cells has been previously reported indicating that the delay in Cx31-GFP removal was not likely due to the presence of GFP [17,30]. Removal of BFA was followed quickly by new assembly of Cx43 gap junctions and reorganization of the Golgi apparatus as denoted by the GM130 staining. Collectively, these studies demonstrate that Cx31-GFP gap junctions are dynamic and generally have a longer life expectancy at cell-cell interfaces than Cx43 gap junctions.

## 4. Discussion

Connexin regulation is exquisitely controlled throughout the rodent epidermis, as no fewer than 7 connexin family members have been reported to be temporally and spatially expressed across the cell layers and differentiation states [8,9,10,11]. These connexin subtypes establish selective and sophisticated communication compartments that are necessary for epidermal barrier function, homeostasis and renewal. In the present study we focus on Cx43 which has been shown to be critical for establishing barrier function in mutant mice [22] and Cx31 that was found to be disposable from genetically-modified mice provided they survived an early placental defect [21]. Fortunately, we have at our disposal an excellent differentiation-competent rat epidermal keratinocyte (REK) cell line that has the capacity to fully mimic rat epidermis when grown at a liquid/air interface [23,27,38]. Since REKs were originally derived from basal keratinocytes, their connexin status mirrors that of cultured primary mouse keratinocytes. The question arose as whether the ablation of Cx43 from these Cx43-rich REKs would potentially destroy all differentiation potential. We already knew the in vivo expression of truncated Cx43 led to a loss of barrier function [22] and Cx43 knockdown greatly impaired the process [23]. We found that differentiation was grossly impaired in Cx43-ablated REKs but not eliminated, further linking Cx43 to the regulation of epidermal homeostasis. Cx31 which is typically found in highly differentiated keratinocytes was observed to assemble into prototypical gap junctions completely independent of Cx43. On the other hand, Cx31 extensively co-assembled into the same gap junctions with Cx30, which are also found in differentiated keratinocytes. Finally, time-lapse imaging and tracking of the fate of Cx31-GFP revealed that these gap junctions were dynamic and long-lived at the cell surface.

Although it is difficult to measure the relative concentration of each connexin isoform in the epidermis due to differences in the efficacy of the antibodies, it is highly probable that Cx43 is the most prominently expressed connexin due to its extensive distribution throughout the epidermis. The fact that Cx43 is enriched in the stratum basale where stem and progenitor cells are programmed to maintain the renewal and homeostasis of the epidermis points to a potentially critical role for this connexin [10]. That said, it is unsurprising that mice harboring a C-terminal truncation mutant of Cx43 fail to establish proper barrier function [22] and keratinocytes with reduced Cx43 levels form partially dysregulated organotypic epidermis [23]. In the present study we show that complete ablation of rat Cx43 led to REKs forming a grossly disrupted epidermis where the living cell layer was thinner, parakeratosis was prominent and keratin 14 was extensively dysregulated. We were surprised that Cx43-ablated keratinocytes still retained considerable differentiation potential but upon observing that they retained substantial GJIC it is possible that other unidentified keratinocyte connexins may have served to maintain their differentiation-competence. These keratinocyte connexins may have been upregulated when stimulated by cellular signals found in more complex organotypic cultures during REK differentiation.

The fact that organotypic epidermis can form when derived from REKs that lack Cx43 expression is in keeping with our understanding of connexin pathologies in humans where Cx43 function can be highly compromised. Patients that carry autosomal dominant missense mutations in *GJA1* (gene that encodes Cx43) clinically exhibit a developmental disorder known as oculodentodigital dysplasia (ODDD), but typically have fully intact skin [43,44,45,46]. Some of these patients even have severely compromised Cx43 function as many Cx43 mutants are dominant to the functional status of co-expressed wild type Cx43 [47,48]. However, Cx43 pathologies can occasionally affect the skin as a set of patients have been identified that harbor *GJA1* mutations and present with erythrokeratodermia variabilis et progressiva (EKVP) [49,50,51]. These patients suffer from transient erythematous patches and hyperkeratotic plaques [49,50,51]. Subsequent studies have assigned hyperactive Cx43 hemichannels as the potential cause of this skin pathology [24,25]. It should be noted that Cx43 gene mutations may evoke a distinctly different phenotypic status compared to complete Cx43 ablation as the mechanisms by which a Cx43 mutant can evoke a disease may include gain-of-function pathological interactions with other connexin family members [48]. Few autosomal recessive nonsense *GJA1* mutations exist in humans. While these patients do harbor many morbidities, they appear to have unaffected skin, at least in the early stages of life [4,43]. Collectively, the role of Cx43 in the epidermis of rodents has overlapping characteristics with Cx43 found in humans but there may also be some subtle differences that have yet to be fully understood.

Cx31 has been understudied since its original cloning in 1991 [20] due in large part to the scarcity of robust and high-fidelity antibodies that can clearly distinguish it from other keratinocyte connexins. Cx31 mouse knockout studies have defined Cx31 as having significant importance in the placenta [21,52,53,54]. However, mice that are born have essentially normal skin even though this connexin is highly expressed in the stratum granulosum [39]. Double Cx31/Cx43 knockout studies further revealed that these connexins may have independent functions in skin development [55]. When expressed in HeLa cells, Cx31 assembled into prototypical gap junctions but these gap junction channels were insensitive to α-glycyrrhetinic acid suggesting they may possess some unique properties compared to some other well studied connexins [56]. In the present study, we further addressed the interplay between Cx43 and Cx31. Ectopic expression of Cx31 revealed that the assembly of Cx31 gap junctions were independent of Cx43 and these connexins rarely co-assembled into the same gap junctions. Conversely, Cx30 and to a lesser extent Cx26, appeared to interact with Cx31 to form mixed gap junctions in keeping with previous FRET findings when these connexins were co-expressed in human NEB1 keratinocytes [8]. Given the limited Cx31 interactome, it was not surprising to see that Cx31 gap junctions were mobile within the plasma membrane undergoing apparent fission and fusion events not unlike what has been well-documented for Cx43 gap junctions [29,34,57,58,59]. It has been known for some time that gap junction plaques are not static structures even though they acquire a semi-crystalline state [60]. It would seem that whether they have a large interactome (e.g., Cx43) with the potential to bind cytoskeletal elements, structural junctions and other stabilizing elements or a small interactome (e.g., Cx31), they retain considerable ability to move within the lipid bilayers of paired cells. However, Cx31 gap junction removal from the cell surface was dramatically delayed compared to Cx43. This is in keeping with pulse-chase studies where Cx31 was found to have a half-life of 6 h when expressed in HeLa cells [56], while Cx43 in cardiomyocytes had a half-life of ~1.5 h [18]. We have previously reported that Cx30 has a prolonged life in keratinocytes compared to Cx43 [19] and it would seem that Cx30 and Cx31 probably share similar life expectancies at the cell surface. The fact that both of these connexins are typically found in highly differentiated keratinocytes may be a clue to their longer survival but this has yet to be established.

Contrary to the case of *GJA1*, *GJB3* (that encodes Cx31) gene mutations in humans often lead to EKVP [50]. As a resident of the stratum granulosum, Cx31 must communicate key molecular signals that prevents the hyperkeratosis and erythematous skin areas associated with EKVP. There are now in excess of 15 *GJB3* mutations associated with EKVP that may act through the generation of endoplasmic reticulum stress or hyperactive hemichannels but this has yet to be fully established as connexin pathologies tend to have multiple molecular mechanisms at their root [25,50]. Interestingly, even though Cx31 is found in keratinocytes where other connexins are almost always present, these connexins fail to sufficiently compensate for the Cx31 defect leading to EKVP. Again, this points to the exquisite sophistication of the epidermis and the critical importance of each keratinocyte connexin.

In summary, we provide unequivocal evidence that Cx43 plays a critical role in proper rodent organotypic epidermis formation, albeit a rudimentary epidermis does indeed form in its absence. Furthermore, Cx31 appears to belong to a subset of keratinocyte connexins with a long residence period at the cell surface as compared to Cx43. Overall, this study continues to inform on the complexity of connexin regulation in the epidermis.
